# Targeting of Topoisomerase I for Prognoses and Therapeutics of Camptothecin-Resistant Ovarian Cancer

**DOI:** 10.1371/journal.pone.0132579

**Published:** 2015-07-24

**Authors:** Yu-Chieh Lee, Chii-Hong Lee, Hsiang-Ping Tsai, Herng-Wei An, Chi-Ming Lee, Jen-Chine Wu, Chien-Shu Chen, Shih-Hao Huang, Jaulang Hwang, Kur-Ta Cheng, Phui-Ly Leiw, Chi-Long Chen, Chun-Mao Lin

**Affiliations:** 1 Graduate Institute of Medical Sciences, College of Medicine, Taipei Medical University, Taipei, Taiwan; 2 Department of Pathology, Taipei Medical University-Shuang Ho Hospital, New Taipei City, Taiwan; 3 Department of Biochemistry, School of Medicine, Taipei Medical University, Taipei, Taiwan; 4 Center for Stem Cells and Translational Cancer Research, Chang Gung Memorial Hospital, Gueishan, Taoyuan County, Taiwan; 5 School of Pharmacy, China Medical University, Taichung, Taiwan; 6 Department of Food and Beverage Management, Taipei College of Maritime Technology, Taipei, Taiwan; 7 Department of Pathology, College of Medicine, Taipei Medical University, Taipei, Taiwan; 8 Department of Pathology, Taipei Medical University Hospital, Taipei, Taiwan; University of South Alabama, UNITED STATES

## Abstract

DNA topoisomerase I (TOP1) levels of several human neoplasms are higher than those of normal tissues. TOP1 inhibitors are widely used in treating conventional therapy-resistant ovarian cancers. However, patients may develop resistance to TOP1 inhibitors, hampering chemotherapy success. In this study, we examined the mechanisms associated with the development of camptothecin (CPT) resistance in ovarian cancers and identified evodiamine (EVO), a natural product with TOP1 inhibiting activity that overcomes the resistance. The correlations among TOP1 levels, cancer staging, and overall survival (OS) were analyzed. The effect of EVO on CPT-resistant ovarian cancer was evaluated *in vitro* and *in vivo*. TOP1 was associated with poor prognosis in ovarian cancers (*p* = 0.024). EVO induced apoptosis that was detected using flow cytometry and terminal deoxynucleotidyl transferase dUTP nick end labeling (TUNEL) assay. The tumor size decreased significantly in the EVO treatment group compared with the control group (*p *< 0.01) in a xenograft mouse model. Effects of drugs targeting TOP1 for prognosis and therapy in CPT-resistant ovarian cancer are anticipated. EVO with TOP1 can be developed as an antiproliferative agent for overcoming CPT resistance in ovarian cancers.

## Introduction

Ovarian cancer has no specific signs or symptoms and is usually diagnosed at an advanced stage, making it the most lethal gynecological cancer in the Western world [1]. Standard chemotherapy for ovarian cancer is frequently accompanied by chemoresistance, which is a major obstacle for the successful treatment of cancer [2] and a second-line chemotherapeutic option must be recommended. Stochastic mutations caused by selection pressure, sequential genetic changes induced by specialized stroma, and stemness of cancer cells were proposed to explain the survival of chemoresistant cells during chemotherapy [3]. Identifying molecular biomarkers for predicting drug sensitivity and resistance is crucial for patient prognoses.

The TOP1 gene copy number, mRNA level, protein level, and enzyme activity are reported to be associated with a poor prognosis in cancer therapy [4]. Establishing the role of TOP1 expression in ovarian cancer may facilitate the development of more effective therapeutic strategies. Notch pathway and DNA topoisomerase I (TOP1) inhibitors have been widely used in cisplatin-resistant ovarian cancers [5]. However, patients can develop TOP1 inhibitor resistance, thus hampering the success of therapy [6]. Use of combination therapy over single-agent chemotherapy leads to improved outcomes in ovarian cancer [7]. Chemotherapy-induced side effects are alleviated after using natural products such as polyphyllin D [8], emodin [9] or Rosa Roxburghii Tratt [10] whereas antitumor activity and immunomodulatory functions are observed [11].

Activation of 5′ AMP-activated protein kinase (AMPK) and subsequent suppression of mTOR activity can induce protective autophagy that confers chemoresistance to tumor cells [12]. Therefore, inhibition of AMPK-associated autophagy can enhance the antitumor effects exerted by chemotherapeutic agents [4,13]. The TOP1 inhibitor camptothecin (CPT) can induce autophagy and reduce apoptosis through the AMPK-TSC2-mTOR pathway in human colorectal cancer [14]. In addition, Topotecan is reported to induce cytoprotective autophagy in wild-type p53 cells but not in mutant p53 or p53 knockout cells [15]. Human A2780 ovarian cancer cells have been analyzed for p53 mutations and classified into a p53 wild-type cell line [16]; therefore, this cell line is expected to easily develop chemoresistance through AMPK-mediated autophagy. This mechanism of action of the Human A2780 cell line provides a potential strategy to screen TOP1 inhibitors with low-protective autophagy activation activity for treating cancers with wild type p53.

Evodiamine (EVO), a quinilone alkaloid, originally isolated from *Evodia rutaecarpa* (Juss.), is reported to possess many physiological functions, including vasorelaxation, antiobesity, anticancer, antibacterial, antiviral, and antiinflammatory effects [17]. Synthetic EVO derivatives have been developed as potent antitumor agents [18]. This study characterized the mechanisms associated with CPT-resistant ovarian cancer cells. The natural product EVO displayed TOP1 inhibitory activity that overcame the CPT resistance of ovarian A2780 cells. Our results provide insights into the increase in the drug susceptibility of CPT-resistant ovarian cancer cells after using EVO-related alkaloids.

## Materials and Methods

### Materials

(*S*)-(+)-CPT (C9911), Evodiamine (E3531), sodium bicarbonate, Dimethyl sulfoxide (DMSO), 3-(4,5-dimethylthiazol-2-yl)-2,5-diphenyltetrazolium bromide (MTT), propium iodide (PI), and Hochest 33342 were purchased from Sigma-Aldrich (St. Louis, MO, USA), Dulbecco’s modified Eagle’s medium (DMEM), trypsin, fetal bovine serum (FBS), L-glutamine, and sodium pyruvate were purchased from Gibco-BRL (Grand Island, NY, USA). An antibody against cyclin D1 was purchased from Ventana (Tucson, AZ, USA). γ-H2A.X, AMPK, phospho-AMPK, acetyl-CoA carboxylase (ACC), phospho-ACC, and glyceraldehyde 3-phosphate dehydrogenase (GAPDH) were purchased from Cell Signaling (Danvers, MA, USA). Ki-67 rabbit monoclonal primary antibody was purchased form DAKO (MIB-5, Denmark). Comet Assay kit was obtained from Trevigen (Gaithersburg, MD, USA). General Layer Medium (GLM) capacity chip was obtained from Bio-Rad (Hercules, CA, USA). The lentiviral vectors (LVs), luciferase (Luc) and green fluorescent protein (GFP) vectors, and beetle luciferin were obtained from Promega (Madison, WI, USA). iView Universal DAB Detection kit was obtained from Ventana (Tucson, AZ, USA). In Situ Cell Death Detection kit, POD, was obtained from Roche Applied Science (Penzberg, Germany).

### Ethics

Human Subject Research: Tissues we used were obtained from Taipei Medical University Hospital and Wan Fang Hospital after Institutional Review Board approval (WFH-IRB-99049). Written informed consent was obtained from all participants. Animal Research: All mouse studies were performed in accordance with guidelines and prior approval of the Institutional Animal Care and Use Committee of Taipei Medical University. IACUC approval No: LAC-99-0302. The animals were humanely killed with isoflurane.

### Specimens

Formalin-fixed paraffin-embedded surgical specimens were used for immunohistochemical (IHC) staining. A histologic diagnosis was performed according to the WHO classification. Tissue microarrays consisted of 180 ovarian surface epithelial carcinomas: 61 serous carcinomas, 35 mucinous carcinomas, 42 endometrioid carcinomas, and 42 clear-cell carcinomas. For the present study, 2 pathologists (Chi Long Chen and Chii-Hong Lee) screened the histological sections and selected areas of representative tumor cells. One tissue core (1.5 mm in diameter) from each specimen was used on detection platforms (Ventana Benchmark GX, Tucson, AZ, USA).

### Cell culture

A2780 was grown in DMEM and CPT-resistant A2780^R2000^ ovarian cancer cells [19] were grown in RPMI1640 that were containing 10% FBS, 4 mM L-glutamine, 4.5 g/L of glucose, 1 mM sodium pyruvate, and 1.5 g/L of sodium bicarbonate at 37°C in a humidified atmosphere containing 5% CO_2_.

### Western blot analysis

Protein samples were resolved using sodium dodecylsulfate polyacrylamide gel electrophoresis (SDS-PAGE) and electrotransferred onto polyvinylidene difluoride (PVDF) membranes, which were incubated with a primary antibody at 4°C overnight, and then incubated with a horseradish peroxidase (HRP)-conjugated secondary immunoglobulin G (IgG) antibody. Immunoreactive bands were visualized using enhanced chemiluminescence (ECL) reagents from PerkinElmer [20].

### Cell viability assay

The cells (10^4^ cells/well) were cultured on a 96-well plate supplemented with culture medium. Cells were treated or untreated for 48 h, and an MTT stock solution (5 mg of MTT/mL of phosphate-buffered saline [PBS]) was added to the growing cultures for 2 h. The absorbance was measured using a spectrophotometer at 560 nm. A blank solution containing only DMSO was considered the reading control [21].

### Flow cytometry

Cells were treated with test compounds for 0–24 h, then trypsinized, pelleted, and washed in PBS. Cell samples were analyzed using a FACS Canto-II flow cytometer (BD Biosciences, San Jose, CA, USA). Annexin-V and propidium iodide (PI) double staining was performed according to the manufacturer’s protocol [22].

### Cell cycle analysis

Cells were seeded at a density of 2 × 10^5^ cells/well in 6-well plates and synchronized by plating in RPMI1640 medium for 24 h for cell cycle analysis. Cells were incubated in 2 mL of complete RPMI1640 with 5 μM EVO and 10 μM CPT for 48 h. After treatment, the cells were harvested with trypsin and washed once with PBS. Before flow cytometry, the cells were incubated with 10 μg/mL of RNase A at 37°C for 15 min. Then, the cells were stained with 5 μg/mL of Hochest 33342 and 20 μg/mL PI for 30 min at room temperature in the dark [23]. Ten thousand cells per sample were analyzed using a BD FACSCalibur flow cytometer (Becton Dickinson, San Jose, CA, USA). The cell cycle was analyzed using MODFIT software.

### DNA relaxation assay

The inhibitory effect of EVO on supercoiled DNA strand breakage caused by TOP1 was evaluated. Plasmid DNA (200 ng) was incubated at 37°C for 30 min in 20 μL of reaction solution (40 mM Tris-acetate, 100 mM NaCl, 2.5 mM MgCl_2_, and 0.1 mM EDTA; pH 7.5) in the presence and absence of a 0–5 μM inhibitor; 0.5 units of TOP1 enzyme were added to start the reaction for 30 min [24]. TOP1-induced DNA strand breakage was indicated by the conversion of the covalently closed circular double-stranded supercoiled DNA to a relaxed form.

### Comet assay (single-cell gel electrophoresis)

To determine the extent of DNA damage of cells, comet assays were performed according to the Trevigen CometAssay kit protocol with slight modifications. A2780^R2000^ cells were treated with TOP1 inhibitors for 1 h. The final cell density was approximately 15,000 cells/mL. The cell suspension was then mixed with low-melting point agarose at 37°C and subsequently transferred to glass slides. Slides were electrophoresed at room temperature and then stained with SYBR Green I for 5 min. On each slide, cell nuclei were examined using a fluorescence microscope. Individual tail moments were calculated using image analysis software (Comet Assay Software Project, http://www.casp.of.pl/). Tail moments were calculated according to the following formula: tail moment = tail DNA% (percentage of DNA in the tail) × tail length (length of the tail) [24]. The mean ± standard error (SE) was calculated from at least 50 cells for each treatment group. Statistical analysis was performed using a 2-tailed unpaired Student’s *t* test.

### Computational molecular docking

The X-ray crystal structure of the human TOP1–DNA complex was retrieved from the Protein Data Bank (http://www.rcsb.org/pdb) for docking studies. After adding hydrogen atoms, the resulting protein–DNA complex structure was used in docking simulations. Chem3D 6.0 software (CambridgeSoft, Cambridge, MA, USA) was used for building the 3D structure of EVO. Furthermore, this structure was optimized on the basis of energy minimization, using the MM2 force field and a minimum root mean square (RMS) gradient of 0.05 Docking simulations were performed using the GOLD program (Version 3.1) on a Silicon Graphics Octane workstation with dual 270 MHz MIPS R12000 processors. The GOLD program uses a genetic algorithm (GA) to perform flexible ligand-docking simulations. The annealing parameters for hydrogen bonding and van der Waals interactions were set to 4.0 and 2.5 Å, respectively. The GoldScore fitness function was applied for scoring the docking poses by using EXTERNAL ENERGY WT = 1.375 [24].

### Analyte assay with a surface plasmon resonance (SPR) sensor chip

Human (h) TOP1 was coupled to the carboxylmethylated dextran surface of a GLM capacity chip according to the protocol described in the Bio-Rad ProteOn One-Shot Kinetics Kit Instruction Manual with slight modifications. Solutions of EVO and plasmid DNA were prepared in a filtered and degassed reaction buffer. All binding experiments were performed at 25°C for a constant flow rate of 100 μL/min of the TOP1 reaction buffer (40 mM Tris-acetate [pH 7.5], 2.5 mM MgCl_2_, 100 mM NaCl, and 1 mM EDTA). The binding affinity of the proteins was evaluated using equilibrium dissociation constants (KD). KD was determined on the basis of a steady-state affinity fitting analysis by using the results from ProteOn Manager 2.0 (Bio-Rad) [24].

### Production of luciferase (Luc)/green fluorescent protein (GFP) A2780^R2000^ cells

For *in vivo* studies, A2780^R2000^ cells were infected with LVs containing Luc or GFP that was driven by a cytomegalovirus promoter. When transplanted into the SCID mice, Luc- or GFP-labeled A2780^R2000^ cells can be monitored using *in vivo* bioluminescence imaging, and GFP-positive cells can be isolated using a flow sorter. Briefly, A2780^R2000^ cells were cultured in 6-well plates such that they attained 20%–40% confluency. A specific titer of the media harvested from LV-producing cells was added to the cultured cells. Plates were centrifuged at 1200 ×*g* for 1 h. Immediately after centrifugation, 2 mL of the specific cell medium was added to each well, and the plates were placed in an incubator. Two days after spinoculation, GFP expression was examined under a fluorescence microscope. GFP-positive cells were sorted using nontransduced cells as a negative control [25].

### Mouse models of labeled tumors

A2780^R2000^ cells (10^6^) were mixed with 500 μL of DMEM and subcutaneously inoculated into 4-week-old SCID mice. After 7 days, the mice were administered intraperitoneal (IP) EVO injections (100 mg/kg) (5 mice per group). The tumor size was measured using calipers every 3 days, and the bioluminescence of the tumors was imaged using a noninvasive IVIS-200 optical system (Xenogen, Alameda, CA, USA) and the Living Image Program (Caliper Life Sciences, Hopkinton, MA, USA). Mice were intraperitoneally administered 300 μL of PBS containing 10 mg/mL of beetle luciferin (Promega, Madison, WI, USA) before they received isoflurane-mediated anesthesia. The quantitative bioluminescence intensity (qBI) was determined as the total photon flux per second [26]. At the end of the experiment, the mice were euthanized, and the tumors were excised and weighed. All mouse experiments were performed in accordance with the guidelines and prior approval of the Institutional Animal Care and Use Committee of Taipei Medical University. All surgery was performed under isoflurane-mediated anesthesia, and efforts were made to minimize suffering.

### Immunohistocytochemistry

Immunohistocytochemical and terminal deoxynucleotidyl transferase dUTP nick end labeling (TUNEL) assays of ovarian cancer cells, harvested at Week 4, were performed according to the manufacturer’s instructions. Formalin-fixed tissues were embedded in paraffin, cut into 4-μm-thick sections, and stained with hematoxylin and eosin (H&E). Immunohistocytochemical staining was performed using Ventana anticyclin D1 (SP4-R), anti-TOP1 (EPR5375), and Ki-67 rabbit monoclonal primary antibodies in a BenchMark ULTRA slide stainer, and samples were analyzed using an iView Universal DAB Detection kit (Ventana Medical Systems). TUNEL staining was performed using the In Situ Cell Death Detection kit, POD (Roche Applied Science), as described previously [27,28]. Positive nuclei were counted in 500 cells, and the number of TUNEL- and cyclin D1-positive cells per high-power field was counted in 5 fields for each coded slide. Expression was analyzed using GraphPad Prism software (Version 5.0; GraphPad Software, San Diego, CA, USA).

### Statistical analysis

Results are presented as the mean ± standard deviation (SD). We used 2-tailed, non-parametric test (Mann-Whitney test) compared with Tumor score and Normal score. Survival curves were analyzed using log-rank Kaplan–Meier (KM) plots. A Cox proportional hazards regression analysis was used to test the prognostic significance of factors in univariate and multivariate models. A *p* value of <0.05 was considered statistically significant. For the comparison of the two grading methods, student’s *t* test statistical analysis was performed.

## Results

### Overexpression of TOP1 is correlated with advanced stage and a poor prognosis in ovarian cancer

**Fig 1:**
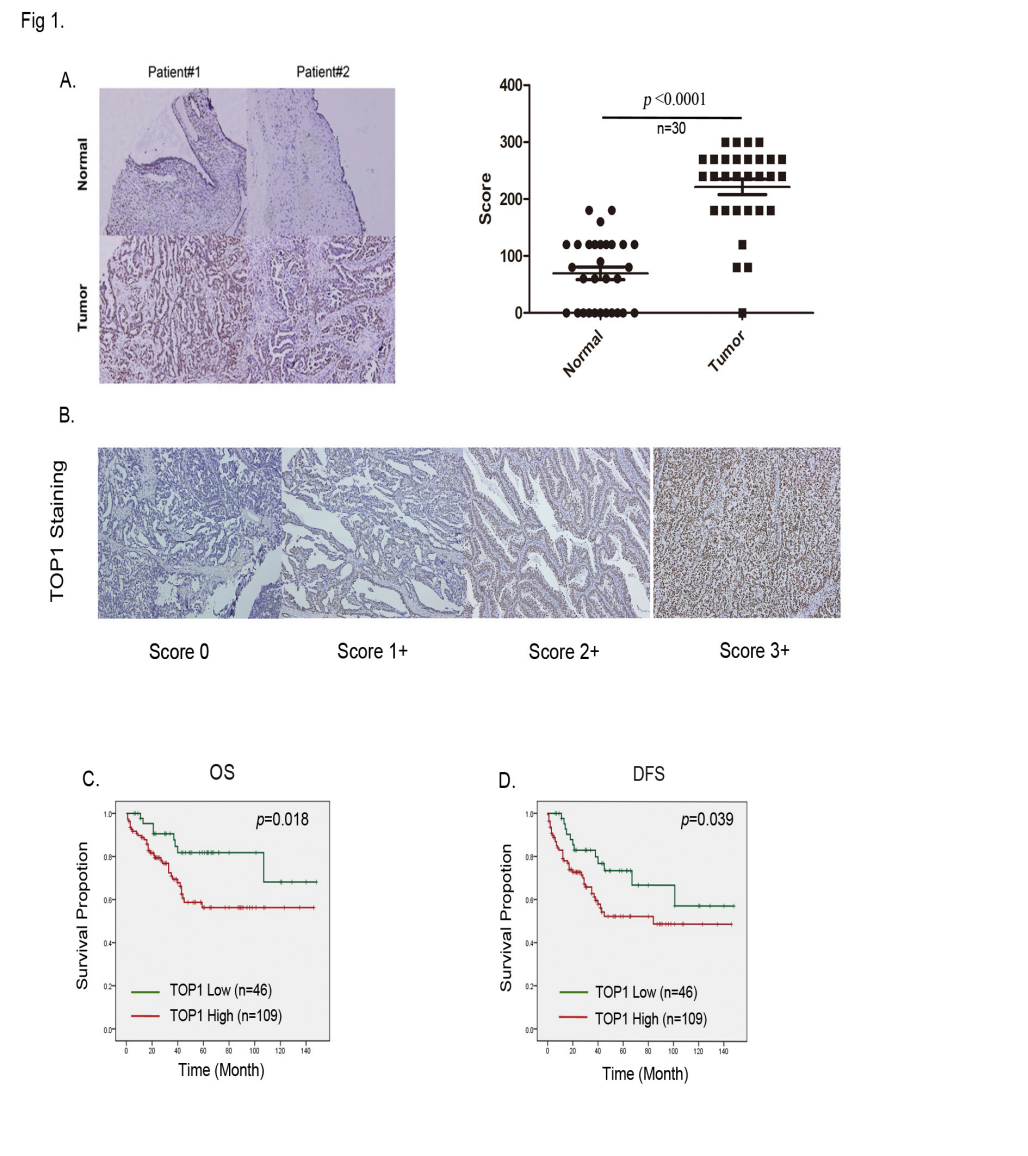
Topoisomerase 1 (TOP1) is overexpression is correlated with an advanced cancer stage and poor survival in ovarian cancer patients. (A) Right, quantification according to nuclear IHC TOP1 expression in ovarian neoplasms compared with paired normal tissues. Left, representative images of IHC staining of cells expressing TOP1 in 30 paired specimens of neoplasm and normal tissues; The scores were calculated using the formula staining intensity × the percentage of stained cells. (B) Scores of 0–3+ indicating TOP1 levels in representative ovarian cancer tissues. (C) The Kaplan–Meier (KM) plot of the overall survival of 155 ovarian cancer patients, stratified on the basis of the TOP1 levels. (D) The KM plot of disease-free survival of 155 patients with ovarian cancer, stratified on the basis of TOP1 levels.

We determined whether TOP1 expression in ovarian cancer was associated with poor survival. Tissues from 156 ovarian cancer patients were examined using IHC staining. The antibody specificity and scoring criteria were defined and scored from levels 0–3 (Fig 1A). We examined 30 sets of matched samples from primary ovarian neoplasm and normal tissues. TOP1 expression was significantly higher in neoplasms than in normal tissues, as evaluated using IHC (Fig 1B, *p* < 0.001). We next investigated the relationship between TOP1 expression levels and the overall survival (OS), defined as patients with a ovarian cancer can die directly from this disease or from an unrelated cause. Overall, 110 of 156 patients had high TOP1 expression levels (with scores of 2+ or 3+), and the remaining 46 patients had low TOP1 expression levels (with scores of 0 or 1+). Furthermore, patients with high TOP1 expression levels had poor OS compared with patients with low TOP1 expression levels. We examined the contribution of the TOP1 mRNA expression to the OS of ovarian cancer patients using the KM plotter (http://kmplot.com/analysis/), which assessed the effect of 22,277 genes on the survival of 1,464 ovarian cancer patients. A background database was established using gene expression data and survival information on 1436 patients that was downloaded from the Gene Expression Omnibus (GEO; Affymetrix HGU133A and HGU133+2 microarrays). The OS analysis revealed that high TOP1 mRNA expression indicated poor survival of ovarian cancer patients (hazard ratio (HR): 1.28, *p* = 0.00062) (S1 Fig). The results showed that high TOP1 expression was significantly associated with a poor prognosis (log-rank *p* = 0.024, Fig 1C). Furthermore, χ^2^ analysis between the TOP1 levels and clinicopathological characteristics of patients showed that TOP1 overexpression in ovarian cancer was associated with the International Federation of Gynecologists and Obstetricians (FIGO) stage (*p* < 0.044; S1 Table). In the univariate analysis, high TOP1 expression (HR: 2.465; 95% confidence interval (CI): 1.139–5.335; *p =* 0.022; Table 1), tumor stage (HR: 8.843; 95% CI: 4.200–18.619; *p <* 0.0001; Table 1) and tumor recurrence (HR: 0.453; 95% CI: 0.243–0.841; *p =* 0.012; Table 1) were all significantly correlated to OS. In addition, Disease Free Survival (DFS), the length of time after treatment during which no disease found, was also associated with TOP1 expression (HR: 1.939; 95% CI: 1.018–3.695; *p =* 0.044), cancer stage (HR: 5.669; 95% CI: 3.143–10.225; *p <* 0.0001), chemotherapy (HR: 1.774; 95% CI: 0.999–3.151; *p =* 0.050), and recurrence (HR: 7.775; 95% CI: 4.515–13.391; *p <* 0.0001). In multivariate analysis, OS was inversely associated with TOP1 expression (HR: 2.290; 95% CI: 1.055–4.971; *p* = 0.036); tumor stage (HR: 0.071; 95% CI: 0.030–0.167; *p <* 0.0001); and chemotherapy (HR: 0.330; 95% CI: 0.151–0.721; *p =* 0.005), and DFS was inversely associate with TOP1 expression (HR: 2.223; 95% CI: 1.150–4.299; *p =* 0.018), cancer stage (HR: 0.150; 95% CI: 0.072–0.310; *p <* 0.001), and recurrence (HR: 7.685; 95% CI: 4.294–13.753; *p <* 0.001; Table 1). In this study, the results revealed an association between TOP1 expression and cancer progression.

**Table 1:** Univariate and Multivariate analysis of TOP1 with regard to OS and DFS.

Variables	Comparison	HR^a^	95% CI^b^	*P*	HR^a^	95% CI^b^	*P*
Cox Univariate analysis (OS)					Cox Multivariate analysis (OS)		
STAGE^c^	Stage12vs.34	8.843	4.200–18.619	<0.0001	0.071	0.030–0.167	<0.0001
Chemotherapy	Yes;No	1.334	0.714–2.492	0.366	0.330	0.151–0.721	0.005
Recurrence	Yes;No	0.453	0.243–0.841	0.012	1.776	0.896–3.521	0.100
TOP1	Low;High	2.465	1.139–5.335	0.022	2.290	1.055–4.971	0.036
Cox Univariate analysis (DFS)					Cox Multivariate analysis (DFS)		
STAGE	Stage12vs.34	5.669	3.143–10.225	<0.0001	0.150	0.072–0.310	<0.0001
Chemotherapy	Yes;No	1.774	0.999–3.151	0.050	0.507	0.240–1.071	0.075
Recurrence	Yes;No	7.775	4.515–13.391	<0.0001	7.685	4.294–13.753	<0.0001
TOP1	Low;High	1.939	1.018–3.695	0.044	2.223	1.150–4.299	0.018

^a^Hazard ratio (HR) estimated from Cox proportional hazard regression model.

^b^Confidence interval of the estimated HR.

^c^TNM system

### EVO binds to TOP1 and inhibits its DNA catalysis activity

**Fig 2:**
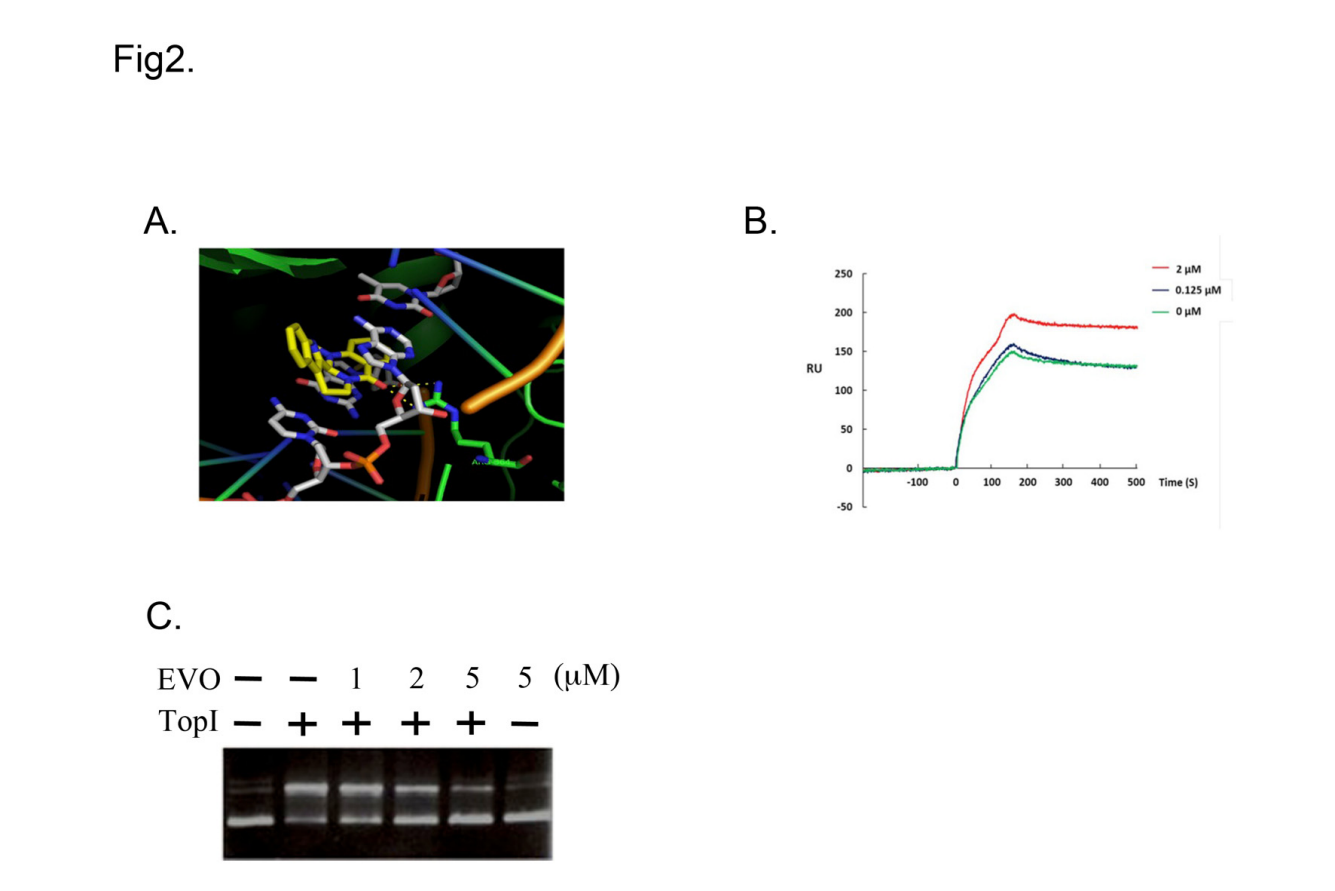
Evodiamine (EVO) interacts with topoisomerase I (TOP1) and inhibits its activity. (A) Computer-aided molecular modeling of EVO docking to TOP1. (B) Surface plasmon resonance sensorgram of the interaction between immobilized recombinant human (h)TOP1 and EVO (0–2 μM). Data are representative of 3 independent experiments. (C) EVO prevented DNA from undergoing hTOP1-induced conversion of supercoiled DNA to relaxed closed circular DNA. pUC19 (0.2 μg) plasmid DNA was incubated at 37°C for 30 min with hTOP1 in the presence of EVO (0–5 μM).

EVO is a TOP1 inhibitor that binds to TOP1–DNA complex sites. A computer-aided molecular model was used to evaluate the docking of EVO to TOP1. Although EVO possesses a nonplanar structure, this compound can intercalate in spaces between the DNA bases to form π-π stacking (Fig 2A). An SPR assay was performed to determine the binding affinity and further characterize drug binding. Recombinant hTOP1 was covalently coupled to the surface of the chip, the combination of pUC19 plasmid DNA (1.0 μg/mL), and EVO (0–2 μM), determined on the basis of the flow rate of the analyte through the sensor chip, and the resonance unit (RU) increased in a concentration-dependent manner (Fig 2B) with a KD value of 6.92 × 10^−22^, calculated using the ProteOn Manager 2.0. The flow rate of the analyte without pUC19 plasmid DNA did not increase the RU. The results account for the binding of EVO to TOP1. To further confirm the hTOP1-inhibitory activity of EVO, we used hTOP1-induced supercoiled PBS (SK+) plasmid relaxation as the assay system. Supercoiled DNA migrated more rapidly on agarose gels than did relaxed circular DNA, as shown in the control (lanes 1 and 2). EVO inhibited hTOP1 catalytic relaxation (lanes 3–5; 1–5 μM) in a concentration-dependent manner (Fig 2C), whereas it alone did not cause change in the DNA topology (lane 6).

### EVO exhibited a cytotoxic effect on CPT-resistant A2780^R2000^ cells

**Fig 3:**
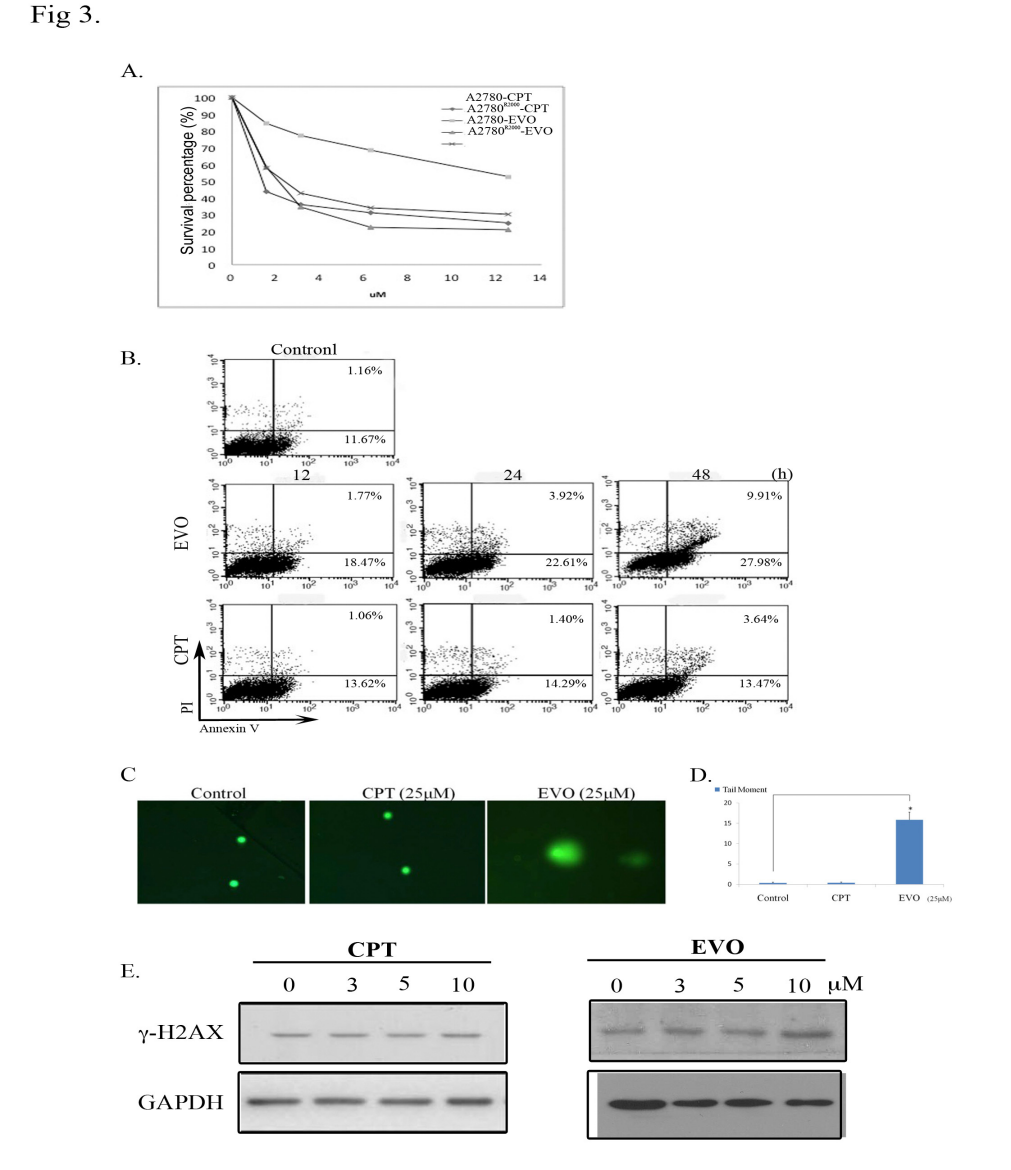
Evodiamine (EVO) exerted a cytotoxic effect on A2780^R2000^ cells. (A) The viability of A2780 and A2780^R2000^ cells was assessed after 48 h of treatment with camptothecin (CPT) or EVO using an MTT assay, and IC_50_ values were determined. (B) FITC/PI flow cytometry of A2780^R2000^ cells treated with EVO (10 μM) for 12–48 h compared with cells treated with CPT (10 μM) for 12-48h. (C). EVO-induced DNA damage in A2780^R2000^ cells. Cells were untreated or treated with CPT and EVO (25 μM) for 1 h and were then analyzed using a neutral comet assay, as described in the Materials and Methods section. Representative images (200×). (D) Histogram of the tail moment plotted against each treatment condition. *p* values of comparisons (marked with *) were <0.05 as determined using a 2-tailed Student’s *t* test. (E) γ-H2A.X levels after EVO or CPT treatment in A2780^R2000^ cells. Cells were treated (0–10 μM) for 6 h, and cell lysates were immunoblotted with an antibody against γ-H2A.X. GAPDH with constant expression was used as the internal control.

We compared the sensitivities of CPT-resistant cells (A2780^R2000^) and parental cells (A2780) to the chemotherapeutic drug, CPT, and EVO. A2780^R2000^ cells exhibited greater resistance to the cytotoxic effects of CPT than that exhibited by A2780 cells (approximately 19-fold high, Fig 3A). EVO exerted similar cytotoxic effects on A2780 and A2780^R2000^ cells with IC_50_ values of 2.18 and 2.38 μM, respectively (Table 2). These data indicate that CPT-resistant ovarian cancer cells are more sensitive to EVO than to CPT. Furthermore, the apoptotic fraction after EVO and CPT treatment was determined using FITC/PI flow cytometry. EVO-triggered apoptosis of A2780^R2000^ cells occurred in a time-dependent manner (20.24% at 12 h, 26.53% at 24 h, and 37.89% at 48 h) (Fig 3B). Treatment of viable cells with TOP1 inhibitors can damage their DNA and elicit responsive repair mechanisms. A comet assay was performed to monitor the EVO-induced DNA damage of A2780^R2000^ cells. EVO treatment (25 μM) for 1 h produced enhanced DNA tailing, indicating increased electrophoretic mobility of DNA fragments. Conversely, the nuclei of control and CPT-treated cells presented a compact round area of fluorescence, with no DNA tail being detected. DNA breaks represented by the tailing area were calculated and compared (*p* < 0.05, vs. untreated cells; using a Student’s *t* test) (Fig 3C and 3D). The phosphorylation of histone H2A.X (γ-H2A.X), a biomarker of DNA double-strand breaks, was detected in cells for further verification. An immunoblot assay was performed to confirm the effect of EVO on γ-H2A.X levels, and the results showed that EVO increased the γ-H2A.X protein levels in a concentration-dependent manner after 6 h of treatment. Compared with CPT treatment, 0–10 μM EVO treatment significantly increased the relative γ-H2A.X levels (Fig 3E). GAPDH with constant expression was used as an internal control.

**Table 2:** IC_50_ (48h) values obtained from MTT assays of CPT and EVO-treated A2700 and A2780^R2000^ cells.

μM	A2780	A2780^R2000^
CPT	0.71	13.57
EVO	2.18	2.38

### EVO induced G_1_ phase arrest and sluggishness of AMPK activation in CPT-resistant A2780^R2000^ cells

**Fig 4:**
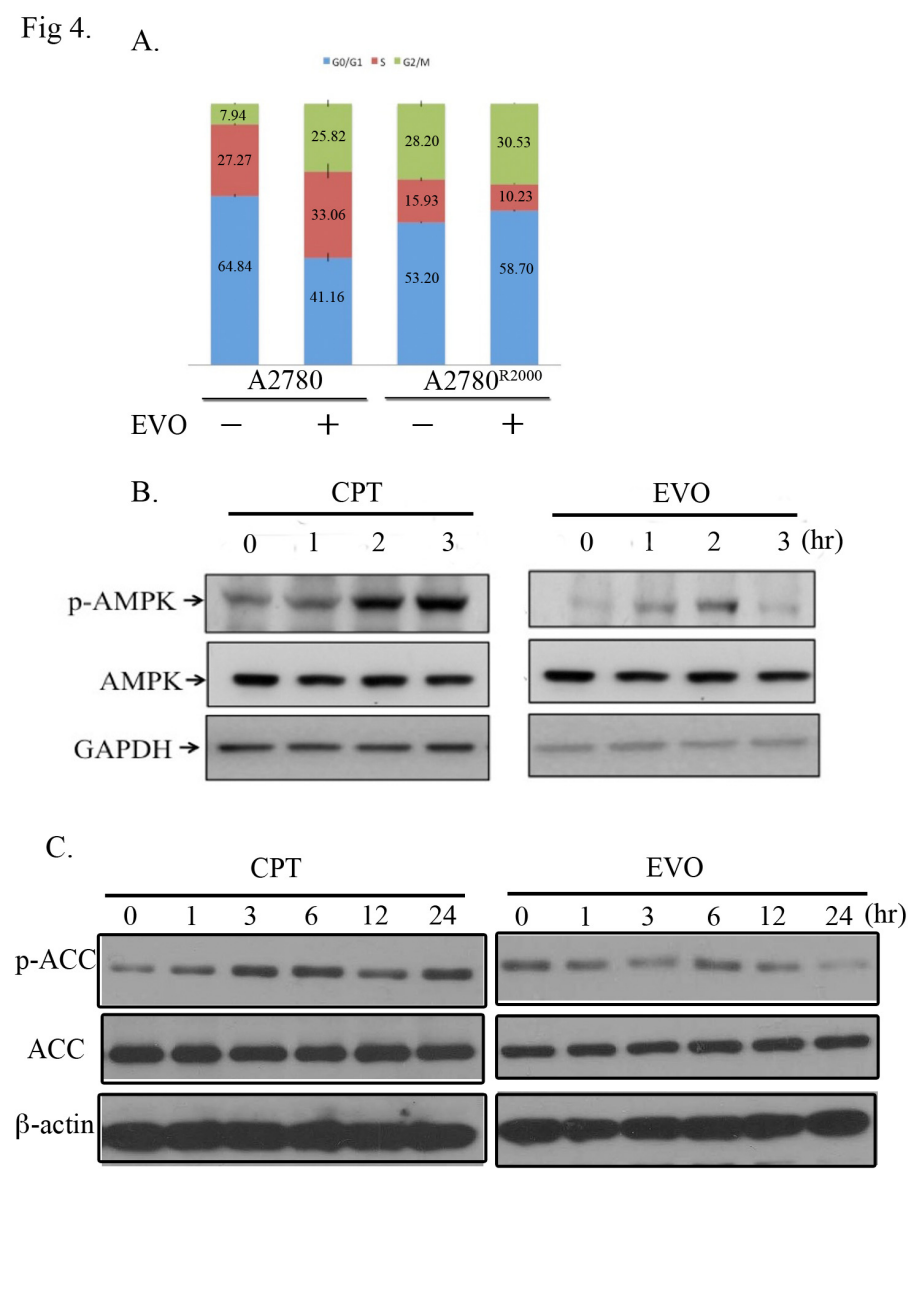
Cell cycles and AMPK activation after evodiamine (EVO) treatments in A2780 and A2780^R2000^ cells. (A) Cell cycle distributions of A2780 and A2780^R2000^ cells after EVO treatment (5 μM) for 48 h. The changes in cell cycle were estimated on the basis of flow cytometry results. Data are representatives of several experiments with similar results. (B) Immunoblot of AMPK phosphorylation after EVO (10 μM) treatment for 0–3 h. (C) Immunoblot of phospho-ACC after EVO (10 μM) treatment for 0–24 h.

Distribution of cells in 3 phases (G_0_/G_1_, S, and G_2_/M) of the cell cycle was determined using flow cytometry analysis, and the proportion for parental A2780 cells was 64.84 ± 0.63, 27.27 ± 0.33, and 7.94 ± 0.36, respectively. EVO treatment (5 μM for 48 h) increased the proportion of cells in the G_2_/M phase (25.82 ± 1.38, *p* < 0.05). The proportion of A2780^R2000^ cells in the G_0_/G_1_, S, and G2/M phases was 53.2 ± 0.36, 15.93 ± 0.66, and 28.20 ± 1.16, respectively. EVO treatment increased the proportion of cells in the G_0_/G_1_ phase (58.7 ± 0.24, *p* < 0.01), whereas no significant change was observed in the proportion of cells in the G_2_/M phase (30.53 ± 0.19) (Fig 4A). EVO treatment induced G_2_/M arrest in parental A2780 cells, whereas it induced loss of G_2_/M arrest and gain of G_1_ arrest in CPT-resistant A2780^R2000^ cells. A difference in cell cycle distribution on CPT (S2 Fig) and EVO treatments was observed in CPT-resistant cells compared with its parental cells. Drug resistance was reported to be associated with enhanced AMPK activity. To determine whether AMPK is activated in A2780^R2000^ cells, the cells were treated with CPT (10 μM) for 0–3 h, and then Western blotting was performed to examine levels of the active phosphorylated form of AMPK (phospho-Thr172). CPT treatment enhanced phospho-AMPK levels in A2780^R2000^ cells (Fig 4B, left). Furthermore, the phospho-AMPK level after EVO treatment for 0–3 h was evaluated. EVO treatment did not elicit persistent phospho-AMPK levels (Fig 4B, right), suggesting that A2780^R2000^ cells exert different responses compared with their parental A2780 cells after CPT and EVO treatments. CPT and EVO treatments revealed consistent changes in AMPK downstream target, phospho-acetyl-CoA carboxylase (ACC) in A2780^R2000^ cells (Fig 4C).

In addition, exposure of A2780^R2000^ cells to 10 μM CPT or EVO for 0–24 h produced different responses in inducing phosphorylation of c-Jun N-terminal kinase (JNK) (Thr183/Tyr185) and extracellular signal-regulated kinase (ERK)1/2 (Thr202/Tyr204) with different timings. JNK phosphorylation started 3 h following CPT treatment and persistently increased up to 24 h, whereas EVO treatment did not increase phospho-JNK. Phosphorylation of ERK started 6 h following CPT treatment and persistently increased to 24 h, whereas EVO treatment did not increase phospho-ERK. Neither CPT nor EVO treatment enhanced the phosphorylation of (Thr180/Tyr182) of A2780^R2000^ cells (S3 Fig).

### EVO inhibited cell proliferation in a xenograft mice model

**Fig 5:**
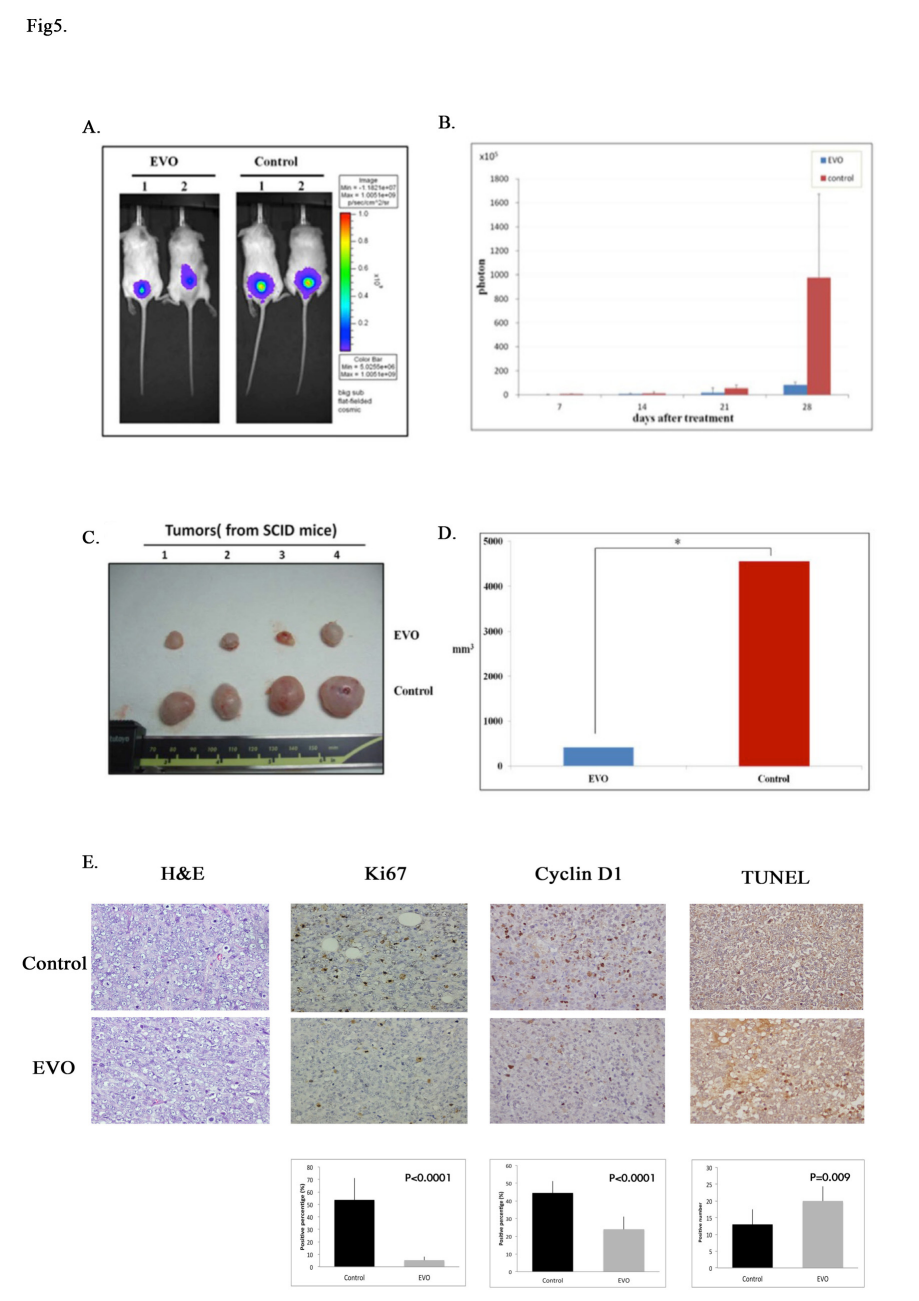
EVO inhibits cell proliferation in a xenograft mouse model. Tumor growth of SCID mice implanted with Luc or GFP expressing A2780^R2000^ cells. (A) Bioluminescence imaging of tumorigenesis in A2780^R2000^ cells in xenograft mice. Statistical results obtained using a Student’s *t* test are shown in (B). (C) Excised tumor tissue of A2780^R2000^ cells in xenograft mice. The statistical results of the excised tumor tissue of A2780^R2000^ cells in the xenograft mice are shown in (D). (E) EVO suppressed Ki-67 and cyclin D1 expression (determined from IHC staining) and resulted in increased apoptosis (TUNEL staining) in tumors. Statistical analysis was performed using GraphPad Prism software and a Student’s *t* test. *** *p* < 0.001.

For *in vivo* studies, A2780^R2000^ cells expressing Luc or GFP genes were isolated and enriched with a flow sorter and then injected into the subcutis of the SCID mice (10^6^ cells/mouse) to establish the ovarian cancer xenograft model. Seven days after cell implantation, the mice were randomly assigned as control or treated groups (100 mg/kg IP EVO treatment). EVO treatment (100 mg/kg) started 7 days after inoculation with A2780^R2000^ cells. Tumor development was periodically monitored on the basis of bioluminescence, measured using the IVIS imaging system, and the tumor size was measured using calipers (S4 Fig). A significant increase in photon intensity was observed in the control group, whereas no such significant increase was observed in the treated group (Fig 5A). The photon intensity in the EVO group (Fig 5B) was significantly reduced compared with that in the control group (*p* < 0.01). Furthermore, the viable tumor volume was determined (Fig 5C). Compared with the control group, a significant reduction in the tumor size was observed in the EVO group (*p* < 0.01) (Fig 5D). Moreover, the mean weights of the excised tumors were lower in mice treated with EVO than in untreated mice. A lower Ki-67 proliferative index, determined using IHC, was observed in mice treated with EVO. To determine whether EVO affected cyclin D1 protein levels and apoptosis of tumor cells *in vivo*, we further analyzed cyclin D1 expression and tumor cell apoptosis in xenograft tumors using IHC and TUNEL staining, respectively. EVO markedly reduced cyclin D1 expression (*p* < 0.001) and increased the number of apoptotic tumor cells (*p* < 0.001) compared with untreated controls (Fig 5E). Consistent with the *in vitro* results, EVO inhibited the growth of A2780^R2000^ cells *in vivo* and suppressed cell proliferation by inducing cell cycle G_1_ arrest and apoptosis.

## Discussion

Surowiak et al. reported a shorter OS time with high TOP1 expression in ovarian cancer patients treated with platinum-based drugs but not with topotecan. Thus, in patients exhibiting high TOP1 expression, application of TOP1 poison-based therapeutic schemes should be considered [29]. Similar results were reported in small-cell lung cancer [30] and melanomas [31]. Only 20%–30% of recurrent colorectal cancer (CRC) patients exhibited an objective response to irinotecan (CPT-11); however, the drug has severe side effects, such as diarrhea, vomiting, neutropenia, and nausea. Assessing TOP1 protein expression as a biomarker for CPT-11 treatment in CRC is crucial to identify patients who are sensitive to CPT-11 treatment [32]. This study revealed that TOP1 expression is associated with a poor prognosis and with tumor progression in ovarian cancers. This finding extends the crucial role of TOP1 as a gynecological marker of TOP1 inhibitor resistance.

Some mutations were identified in hTOP1 DNA as conferring resistance to CPTs. The crystal structures of mutated hTOP1 in ternary complexes with topotecan and DNA were established, and they provided insights into the resistance mechanisms. Computational simulations were used to investigate the molecular principle of TOP1 inhibitor resistance. Mutations of E418K, G503S, and D533G were reported to play critical roles in the development of drug resistance. A structural analysis can provide valuable clues for designing improved inhibitors that combat resistance [33]. DNA TOP1 of A2780^R2000^ has mutations at G717V and T729I amino acid residues that exert a synergetic effect on CPT resistance by targeting the catalytic site of the enzyme–DNA complexes [19]. Results of the structure-based molecular modeling facilitated the understanding of the binding of EVO to the TOP1–DNA complex; the binding site of this docking was consistent with that observed by Yu *et al*. [34]. Pan *et al*. identified EVO as a catalytic inhibitor of TOP1, rather than as stabilizing TOP1-DNA-EVO ternary cleavage complexes in leukemia cells [33]. EVO demonstrates its ability to overcome the resistant binding that was observed for CPT and exerts cytotoxic effects on A2780^R2000^ cells, suggesting that its binding mode differs from that of CPT. In this study, we examined the use of EVO as a TOP1 inhibitor that counters drug resistance.

Activation of sustained AMPK activity in cells exposed to anticancer drugs leads to increased chemoresistance, eliciting autophagy that can protect cells from death in response to anticancer drugs [27,35]. However, Matrone *et al*. proposed a strategy of promoting activation of AMPK/FoxO3A signaling against ovarian cancer [36]. Autophagy was correlated with the severity of oxidative stress [28]. The role of autophagy in the cellular response to therapeutic drugs is controversial. Several studies have reported that apoptosis and autophagy can concomitantly occur in the same cells under certain circumstances. Accumulating evidence suggests that complex interrelationships exist between the autophagic and apoptotic cell pathways. In this study, CPT-treated A2780^R2000^ cells exhibited sustained AMPK phosphorylation that resulted in cytoprotection, whereas EVO treatment reduced the sustained AMPK phosphorylation, resulting in enhanced cell death. AMPK-associated autophagy is reported to remove damaged mitochondria and prevent apoptosis by blocking the release of proapoptotic substances from mitochondria [37]. Accordingly, it is speculated that autophagosomes promote the digestion of the released apoptosis-inducing factors and lead to cell survival by reducing CPT-triggered programmed cell death.

DNA damage checkpoints work throughout the cell cycle to maintain genetic integrity by preventing damaged DNA replication or segregation. Inhibition of cyclin–cyclin-dependent kinase (CDK) complexes to delay cell cycle progression plays critical roles in checkpoint actions [38]. EVO is able to inhibit the proliferation of human gastric cancer cells [39], which is associated with apoptosis, autophagy, and cell cycle arrest at the G_2_/M phase [40]. However, EVO initiated atypical apoptosis in fibroblasts and breast cancer cells by inducing cell cycle arrest at the G_0_/G_1_ phase [41] with reduced amounts of Bcl-2, cyclin D1, and CDK [42]. EVO has a higher capacity than single-target drugs to counter tumor metabolism and arrest tumor development. In this study, EVO arrested the G_2_ phase in parental A2780 cells and the G_1_ phase in CPT-resistant A2780^R2000^ cells. The EVO targets for G2/M arrest in parental A2780 cells seemed to decline along with the development of CPT resistance and resulted in the arrest of the G1 phase. TOP1 mutation caused weaker TOP1–DNA trapping by CPT, thus enabling tighter trapping by EVO and reduced cyclin D1 expression and resulting in G_1_ arrest in A2780^R2000^ cells. The ataxia-telangiectasia mutated (ATM) product is essential for TOP1 poison-induced checkpoint responses, DNA double-stranded breaks (DSBs), and cell cycle arrest through p53 activation and DNA repair [43]. ATM-defective cells are sensitive to DSB-inducing agents, making ATM a possible target for EVO action in A2780^R2000^ cells. By comparing the sensitivity of parental A2780 cells with the CPT-resistant derivative, we found that EVO possesses TOP1-poisoning activity in A2780 ^R2000^ cells. EVO showed a robust potential for CPT resistance-targeting therapy.

Differential cyclin D1 expression, a protein essential for the G_1_ to S progression of the cell cycle, plays a crucial role in malignant diseases [44]; and cyclin D1 overexpression has been reported in ovarian cancer [45]. Cyclin D1 affects the epithelial-to-mesenchymal transition (EMT) in ovarian cancer stem cells [46], which is crucial in chemoresistance to therapy because of the ability of cells to contribute to cancer invasion and metastasis [2]. EVO exerts cytotoxic effects on breast cancer cells, and the effects include cell cycle arrest, a decrease in cyclin D1 expression, and induction of cell apoptosis [42,47]; Furthermore effects of EVO on human hepatocellular carcinoma [48] and gastric tumors have been proven [49]. In this study, EVO exerted its activity by downregulating the cyclin D1 protein expression and inducing apoptosis in a xenograft mouse model, in which CPT-resistant ovarian cancer cells were transplanted. Our current results could facilitate the development of EVO as an antiproliferative agent for CPT-resistant ovarian cancers.

In conclusion, this study demonstrates that DNA topoisomerase I is a reliable marker for ovarian cancer prognosis. The ovarian cancer cells resistant to CPT are associated with AMPK and MAPK activity, and evodiamine, a topoisomerase I-targeting natural product, is effective in combating CPT-resistance in ovarian cancer cells.

## Supporting Information

S1 Fig Expression of TOP I is correlated with survival in patients from KM plotter in ovarian cancer. Kaplan-Meir survival analysis of TOPI in 1464 patients with ovarian tumors. Auto select best cutoff was chosen in the analysis; Cutoff value used was 1078; Expression range of the probe was 12–7625.
(JPG)

S2 Fig Cell cycle distributions of A2780 and A2780^R2000^ cells after CPT treatment (10 μM) for 48 h. (TIF)

S3 Fig Evodiamine (EVO) and camptothecin (CPT) treatments induced different MAPK responses in resistant ovarian A2780^R2000^ cells. (TIF)

S4 Fig Treatment of Evodiamine suppresses the growth of A2780^R2000^ cell xenografts in vivo. (JPG)

S1 Table Association of TOP1 expression with clinicopathological features. (DOCX)
