# Supplementary figures and images for: Targeting of Topoisomerase I for Prognoses and Therapeutics of Camptothecin-Resistant Ovarian Cancer
Source: PLoS One. 2015 Jul 24;10(7):e0132579. doi: 10.1371/journal.pone.0132579 (PMC4514822; doi:10.1371/journal.pone.0132579)

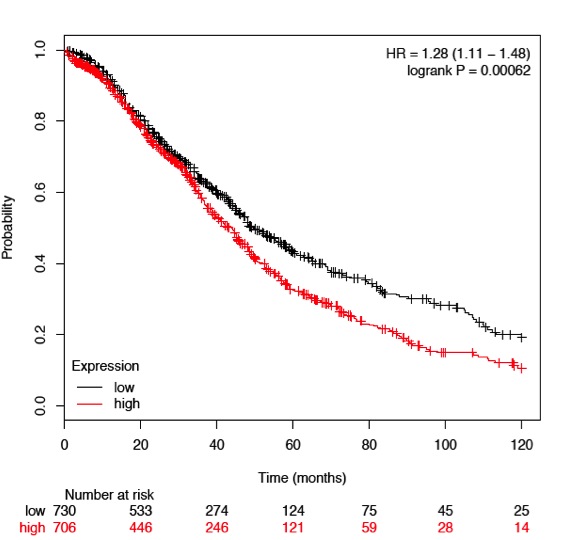

Supplement: S1 Fig — Kaplan-Meir survival analysis of TOPI in 1464 patients with ovarian tumors. Auto select best cutoff was chosen in the analysis; Cutoff value used was 1078; Expression range of the probe was 12–7625. (JPG) [file pone.0132579.s001.jpg]

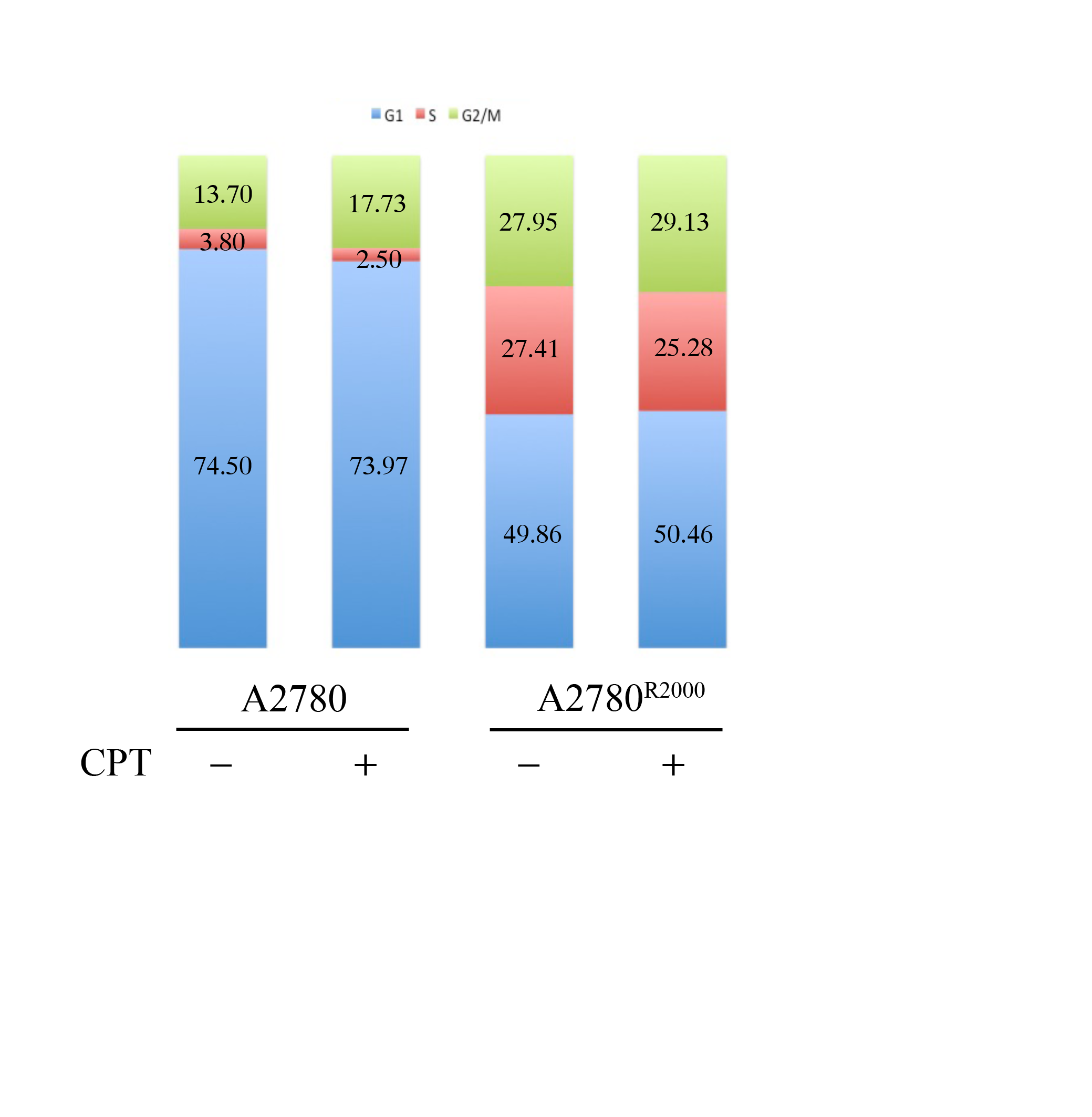

Supplement: S2 Fig — (TIF) [file pone.0132579.s002.tif]

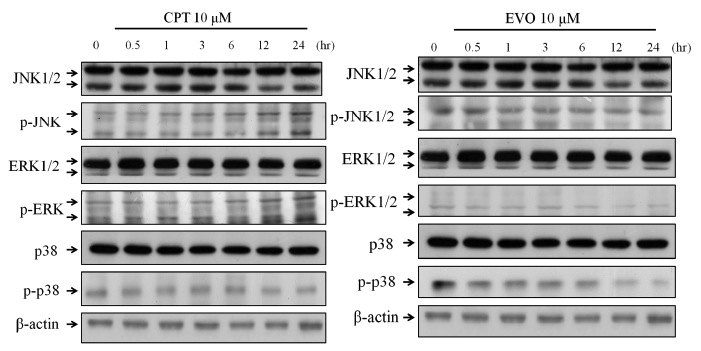

Supplement: S3 Fig — (TIF) [file pone.0132579.s003.tif]

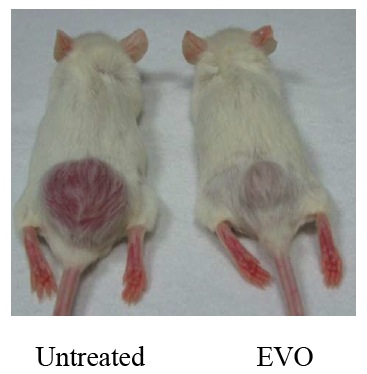

Supplement: S4 Fig — (JPG) [file pone.0132579.s004.jpg]
