# Supplementary material for: Targeting of Topoisomerase I for Prognoses and Therapeutics of Camptothecin-Resistant Ovarian Cancer
Source: PLoS One. 2015 Jul 24;10(7):e0132579. doi: 10.1371/journal.pone.0132579 (PMC4514822; doi:10.1371/journal.pone.0132579)
Supplement: S1 Table — (DOCX) [file pone.0132579.s005.docx]

| **Clinical factor** | **TOP1 low** | **TOP1 high** | ***P*** |
| --- | --- | --- | --- |
| **Type** |  |  |  |
| Clear carcinoma | 9(19.6) | 23(21.1) | 0.980 |
| Endometrioid carcinoma | 12(26.1) | 25(22.9) |  |
| Serious carcinoma | 15(32.6) | 40(36.7) |  |
| Mucinous carcinoma | 9(19.6) | 19(17.4) |  |
| Others | 1(2.2) | 2(1.8) |  |
| **FIGO** |  |  | 0.044 |
| I | 25(54.3) | 53(48.6) |  |
| II | 4(8.7) | 12(11) |  |
| III | 11(23.9) | 41(37.6) |  |
| IV | 6(13) | 3(2.8) |  |
| **Chemotherapy** |  |  | 0.866 |
| NO | 20(43.5) | 49(45) |  |
| YES | 26(56.5) | 60(55) |  |
| **Recurrence** |  |  | 0.688 |
| NO | 38(82.6) | 87(79.8) |  |
| YES | 8(17.4) | 22(20.2) |  |
| **Live** |  |  | 0.062 |
| NO | 38(82.6) | 8(17.4) |  |
| YES | 74(67.9) | 35(32.1) |  |

S1 Table. Association of TOP1 expression with clinicopathological features
